# Cocaine in Cautery of Venereal Warts

**Published:** 1887-07

**Authors:** D. Berry

**Affiliations:** President West Texas Medical Association, San Antonio, Texas


### COCAINE IN CAUTERY OF VENEREAL WARTS.

*By D. Berry, M. D., President West Texas Medical Association, San Antonio, Texas.*

For Daniel's Texas Medical Journal.

I WOULD like to draw the attention of the profession to the efficacy of cocaine as a local anæsthetic in the cauterization of venereal sores, and of the prompt and effectual action of iodoform in cases of ulceration where screw worms have taken up their abode. We read constantly in the medical literature of the day where cocaine is used in almost every manner of surgical procedure, but I do not think in a single instance have I read of its being used in connection with the cauterization of venereal sores.

On May 25, I was called to see Mr. T., a railroad man, who told me that he had an immense syphilitic sore, which was making rapid progress, that he desired to consult me about. On examination, I found a most obstinate case of phymosis, which I eventually, however, succeeded in retracting behind the glands, when a large phagedenic chancroid presented itself. I determined at once to cauterize it with fuming nitric acid. The patient had suffered similarly a few years before, and having had some experience with nitric acid then, expressed himself as being unable to stand the pain without an anæsthetic. Not having anyone at hand to administer chloroform, and happening to have a four per cent solution of cocaine, I determined to try its properties in a case of this kind

After applying the anæsthetic for five minutes, I used the fuming nitric acid thoroughly to the phagedenic surface without the patient experiencing the slightest pain. The surface was afterward dressed with powdered iodoform, and such constitutional treatment employed as was indicated by the patient's general condition. At the present writing the sore has nearly healed, and the man in a fair way to make a speedy recovery.

Case No. 2, is one of the most loathsome that it has been my misfortune so far to encounter. On the morning of June 2, I was called to see a woman whose genital organs, the messenger told me, were filled with screw worms. On arriving at the house I found a woman 65 years of age who had been sick for several months, and who was at the time I saw her sinking rapidly. On making an examination per vaginum, it was found to be filled as far as I could see with a moving mass of worms; they were also crawling from the anus. On introducing a rectal speculum the same state of affairs was found to exist in the rectum. The mucous membrane of the vagina and rectum was reached and bathed in a muco-purulent discharge. I cleared away as many of the parasites as I could with a warm water injection, and swabbed the parts thoroughly with a mixture containing ℥ii of iodoform to ℥ii of glycerine, and instructed the nurse in the course of 2 or 3 hours to give a warm water injection. On my visit in the evening I was told by the nurse that the injection had brought away a large quantity of dead worms.

When I examined the vagina and rectum, they were found to be perfectly free of the parasites. The woman however was in *extremis*, and died during the night.

I have had several cases where screw worms were found, and before experimenting with iodoform tried calomel, bi. chlor. mercury and ac. carbolic, with only partial results. Iodoform kills them almost instantly, and in the treatment of such cases I regard it as *the* remedy *par excellence*.

